# Polymeric Membranes for Biomedical Applications

**DOI:** 10.3390/polym15030619

**Published:** 2023-01-25

**Authors:** Elena Ruxandra Radu, Stefan Ioan Voicu, Vijay Kumar Thakur

**Affiliations:** 1 Department of Analytical Chemistry and Environmental Engineering, University Politehnica of Bucharest, 011061 Bucharest, Romania; 2 Advanced Polymers Materials Group, University Politehnica of Bucharest, 011061 Bucharest, Romania; 3 Biorefining and Advanced Materials Research Center, Scotland’s Rural College (SRUC), Kings Buildings, Edinburgh EH9 3JG, UK; 4 School of Engineering, University of Petroleum & Energy Studies (UPES), Dehradun 248007, Uttarakhand, India; 5 Centre for Research & Development, Chandigarh University, Mohali 140413, Punjab, India

**Keywords:** polymeric membranes, biomedical applications, hemodialysis, drug delivery, artificial organs, tissue engineering

## Abstract

Polymeric membranes are selective materials used in a wide range of applications that require separation processes, from water filtration and purification to industrial separations. Because of these materials’ remarkable properties, namely, selectivity, membranes are also used in a wide range of biomedical applications that require separations. Considering the fact that most organs (apart from the heart and brain) have separation processes associated with the physiological function (kidneys, lungs, intestines, stomach, etc.), technological solutions have been developed to replace the function of these organs with the help of polymer membranes. This review presents the main biomedical applications of polymer membranes, such as hemodialysis (for chronic kidney disease), membrane-based artificial oxygenators (for artificial lung), artificial liver, artificial pancreas, and membranes for osseointegration and drug delivery systems based on membranes.

## 1. Introduction

The biomedical field is a transdisciplinary field that combines knowledge from medicine, biology, biochemistry, materials science, and biomechanics. A considerable part of biomedical fields is represented by the development of biomedical applications, such as the evolution of biocompatible implants and other medical devices, imaging equipment, regenerative tissue engineering, and drug delivery systems [1]. Biomedical devices, such as bone implants, contact lenses, stents, artificial hearts, tissue adhesives, surgical sutures and dialysis membranes, are obtained by using biomaterials [2,3,4,5]. Biocompatibility is an important property when it comes to the materials used in biomedical applications because it is necessary to use some friendly materials, which are able to not harm the living tissue [6]. Further, the testing of biocompatibility through *in vitro* and *in vivo* specific tests is an essential step in developing biomedical devices [7].

**Figure 1:**
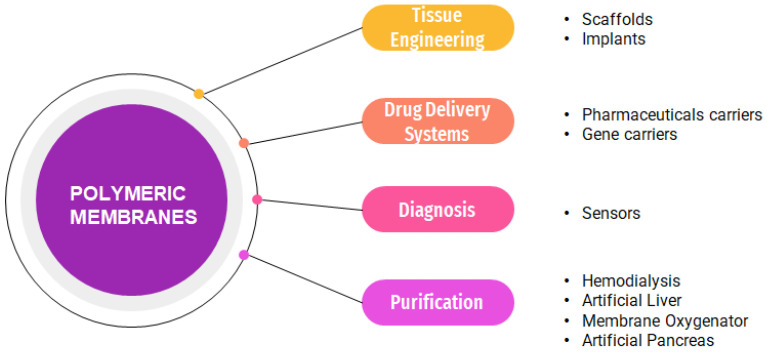
Polymeric membrane applications in the biomedical field.

In recent years, the use of membranes in various medical applications has been constantly evolving [8,9,10,11,12,13]. Membranes are defined as porous films that act as a separating barrier between two adjacent phases, capable of allowing the transport of substances from one phase to another in a selective manner [14,15]. In general, membranes utilized in the biomedical field can be used in tissue engineering, with a role in purification and in obtaining implants and scaffolds, in obtaining controlled release systems of active substances and in diagnosis, in the form of sensors and various diagnostic tests [16]. Membranes are classified according to their nature into natural membranes and artificial membranes. Synthetic membranes can be further classified according to the material into organic, inorganic or composite membranes [17]. Organic membranes are generally obtained from natural polymers such as cellulose, chitosan, collagen and alginates or from synthetic polymers such as poly (ethylene glycol) (PEG), polyurethane, polysulfone, polylactic acid, poly (acrylic amide), poly (N-vinyl-2-pyrrolidone) [18,19,20,21,22,23,24]. In the biomedical field, membranes are used to detoxify the blood. A well-known example is represented by the membranes used in hemodialysis, in cases of end-stage renal disease, where part of the kidney function is replaced by using a flat membrane or empty fiber dialyzer to remove excess water, salts and excreted metabolic products [24]. However, membranes are often used in other biomedical applications, such as in the developing of artificial organs (artificial liver, oxygenator and pancreas) for increasing the optimal functionalization of physiological functions of the organs [25,26,27]. Further, the application of polymeric membranes in tissue engineering has been highly studied due to their biofunctionality, good mechanical properties and the ability of a possible reparation and regeneration of injured tissues/organs [28,29,30,31,32,33]. Another application of polymeric membranes in the biomedical field is represented by the developing drug delivery system based on membranes [34] or different separation interest molecules such as antibiotics [35,36,37] or proteins [38]. The release of the drug is achieved by the diffusion of the active substance through the polymeric membrane so that the drug release could be controlled and targeted [39,40,41]. This article presents the recent developments of polymeric membranes in biomedical applications (Figure 1).

## 2. Biomedical Applications of Membranes

### 2.1. Hemodialysis

Renal failure is one of the major problems suffered by >850 million people worldwide [42]. In chronic kidney failure disease, the kidney functions of the patients are deteriorated, leading to the incapacity of filtering the blood by removing waste products from the body, such as metabolic-resulted toxins with molecular weights more equal to less than 58 kDa [43,44]. Generally, hemodialysis is used in order to ensure a better quality of life for patients suffering from chronic kidney failure [45]. The US Data Renal System (USRDS) reports that almost 80% of hemodialysis patients started dialysis using an indwelling catheter [46]. Further, hemodialysis is defined as an extracorporeal blood purifying method using a semipermeable membrane to conduct blood purification and remove uremic toxins [44,45]. The principle of the hemodialysis membrane is the diffusion of the solvent through a semipermeable membrane [47]. The hemodialysis treatment is used to remove small molecules such as small water-soluble toxins (molecular weight, MW < 500 D), but also a small amount of the middle molecules (MW 500–32,000 Da) from the blood [48]. In hemodialysis treatment, the patient is connected to a dialysis machine and their blood is pumped out via vascular access and filtered using a dialyzer, which is called an artificial kidney. The filtered blood is then pumped back into the patient’s body [49].

The major drawbacks of using semipermeable membranes in hemodialysis are the hemocompatibility through blood exposure to the membrane’s material, which could lead to activation of proinflammatory molecules, and the incapacity of successfully removing some larger toxins molecules [44]. The development of upgraded hemodialysis membranes for increased hemocompatibility and anticoagulant properties was reported [44,50,51,52,53,54]. These membranes were obtained from natural or synthetic polymers, such as polysulfone (PSF) [55,56,57], polyethersulfone (PES) [58,59], polyvinyl alcohol (PVA) [60,61,62], cellulose triacetate (CTA) [63,64,65], polymethylmethacrylate (PMMA) [66,67], polyacrylonitrile (PAN) [53,68], and polyamide (PA) [67].

**Figure 2:**
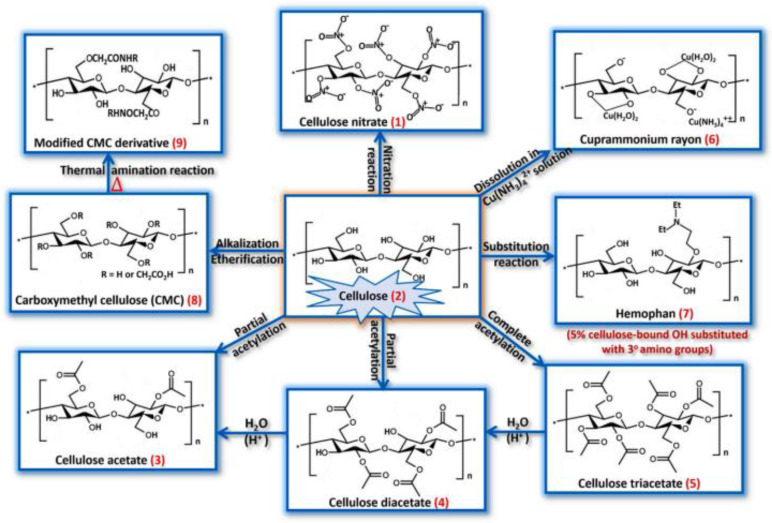
Molecular structures of cellulose and some cellulose derivatives (reproduced with permission after [65]).

The first used hemodialysis membranes were made from cellulose-based membranes. Cuprophan® (Wuppertal, Germany) was the first used cellulose-based hemodialysis membrane that was obtained from cotton [48]. Nowadays, cellulose-based hemodialysis membranes are obtained via acrylation with acetate groups resulting in cellulose acetate, cellulose diacetate (CDA), and cellulose triacetate (CTA) with free hydroxyl groups on the surfaces [48,69]. Figure 2 presents the main cellulose-based derivates [65]. The cellulose-based hemodialysis membranes have good toughness, biodegradation, sustainability, and biocompatibility and are cheap in comparison to other polymers [70]. Cellulose acetate (CA) is the most utilized cellulose derivate due to its great solubility in diverse organic solvents and insoluble in water. Dumitriu et al. [63] showed CA functionalization with TiO_2_ nanoparticles, followed by heparin incorporated via dopamine polymerization. The contact angle results showed that both the neat CA membrane and composite membrane present a hydrophilic nature, but a slight decrease in the contact angle for the composite membrane was observed. Faria et al. [71] studied the potential uremic blood purification with cellulose acetate (CA) functionalized with SiO_2_ nanoparticles. It was observed that at higher SiO_2_ content (18%) the hydrophilicity of the membrane increases. The hydraulic permeability increases after the addition of SiO_2_ nanoparticles, and the rejection of BSA was 99%. Azhar et al. [60] presented the CA-based membrane modified with PVP for better hemocompatibility. The results showed increased hydrophilicity, the pure water flux, BSA rejection, urea, and creatine clearance obtained is 42.4 ± 2 L/m^2^h, 95 ± 1.023%, 93 ± 1.023% and 89 ± 1.023%. 

Polysulfone (PSF) is the most used material in the fabrication of the hemodialysis membrane, with almost 90% of hemodialysis membranes being made of PSF and PSF derivates [72]. PSF is a thermoplastic polymer with great mechanical properties, thermal stability, biocompatibility, and water permeability [47,73]. The PSF membrane showed great stability in extreme basic or acidic environments [74]. Additionally, PSF membranes are resistant to sterilization with steam, ethylene oxide, and gramma radiation [75]. The main disadvantage of PSF is its hydrophobic nature, which favors the molecule adhesion on the membrane surface, inducing blood clot formation [76]. In the past years, researchers investigated the functionalization of PSF in order to increase surface hydrophilicity, resulting in improved hemocompatibility and antifouling properties [54,77]. Polyether sulfone (PES) has similar properties as PSF, with great oxidative, thermal and mechanical properties [78]. The same as PSF, the limitation of PES is represented by the hydrophobic nature, which affects the hemocompatibility of the membrane, even if it is more hydrophilic than PSF due to the presence of the higher atomic weight ratio of sulfone groups [79]. The biocompatibility of neat PES isn’t satisfactory enough and in the past years, researchers obtained different PES-based membranes by adding different additives or by functionalizing the pristine PES. Irfan et al. [80] proposed membrane composites based on PES, multi-wall carbon nanotubes (f-MWCNT) and polyvinylpyrrolidone (PVP) via the phase inversion process for hemodialysis application. The contact angle of the nanocomposite membrane was significantly lower than the PES contact angle (from 88° for PES to 51° for nanocomposite). Further, pure water permeation flux (PWP) rate up to 72.20 L m^-2^ h^-1^ exhibited 58.82% reduced protein absorption and better uremic waste clearance of 56.30%, 55.08% and 27.90% of urea, creatinine and lysozyme. Abdelrasoul et al. [52] proposed PES-PVP composite membrane via UV-assisted photochemical synthesis. The addition of PVP increased the hydrophilicity of the composite membrane and showed better resistance to fibrinogen adsorption compared to the pristine PES membrane. Hoseinpour et al. [81] presented carboxymethylcellulose (CMC), and SCMCs were immobilized on the surface of the aminated PES membranes (PES-NH2) via amide bonds. The hydrophilicity of composites was increased in comparison with the hydrophilicity of neat PES due to the surface functional groups of NH_2_ in PES-NH_2_, COO− in PES-CMC and COO− and SO_3_− in PES-SCMC membranes engaging in hydrogen bonding interactions with H_2_O. The bovine serum albumin (BSA) rejection increases in the case of the composite’s membrane up to 99% in comparison with pristine PES (95%). Xing et al. [82] described the antimicrobial properties of polydopamine (PDA) and PES composites to immobilize antimicrobial metal ions with their functional groups. The hydrophilicity increases after the addition of PDA such that the contact angle decreases from 102° for PES to 40° for the composites.

Generally, the addition of polyvinylpyrrolidone (PVP) is used as an additive in order to increase the hydrophilicity of PES and PSF membranes [83]. In membrane synthesis via phase inversion, PVP plays a major role in the pore formation of the membrane [84]. Mansur et al. [85] presented the effect of the addition of different PVP concentrations (3 wt% and 18 wt%) on the PSF membrane. It was observed that at a higher concentration of PVP, the membrane pores were larger than after the addition of a lower PVP content, leading to increased hydrophilicity of the membrane and also leading to an increased flux and permeability of the membrane, but the BSA rejection was significantly lower at higher concentrations of PVP.

Researchers had proposed surface functionalization with different anticoagulant agents, such as heparin or heparin-like molecules, for better hemocompatibility. Heparin is an anticoagulant agent used in medicine for the prevention of thrombus formation and limits thrombus extension [86]. Heparin has been immobilized onto membranes to increase hemocompatibility through physical absorption or ionic bonding [87,88,89]. Huang et al. [90] presented the chemical binding of heparin onto PSF sheets via a three-step synthesis method (Figure 1). A decrease in the contact angle after functionalization was observed, leading to increased hydrophilicity and hemocompatibility. Ren et al. [91] described the PSF functionalization of heparin via covalent immobilization in order to improve anticoagulant properties. After functionalization, the contact angle decreased from 87° to about 30^o,^ and the coagulation time was prolonged with less fibrin generated in the process of hemodialysis. The limitations of heparin treatment are the expensive cost and potential side effects [92,93]. 

Different molecules were studied in order to replace the addition of heparin. The heparin-like molecules present an alkyl backbone for synthesized polymers or a uronic backbone for modified biomacromolecules [94]. Zhang et al. [95] used tannic acid as a heparin-like substitute to increase the anticoagulant properties of the PES membrane. The BSA rejection rate and urea clearance rate were 97.1% and 92.0% after the addition of tannic acid. Additionally, the hydrophilicity of the composites reduced platelet adhesion and activation, improving the PES membranes’ hemocompatibility. Tannic acid is a bio-based polyphenol that can be found in all aerial plant tissues [96]. Ma et al. [94] proposed sodium alginate as a heparin-mimicking molecule. The results showed that the presence of sulfonic groups and uronic main chain structure gives excellent anticoagulant activity. Lu et al. [97] proposed heparin-like anticoagulant polypeptides due to the carboxyl and hydroxyl side groups for anticoagulant and thrombolytic therapy. In addition, by simply adjusting the feeding ratio of monomers, anticoagulant activity can be regulated. Song et al. [87] proposed carboxymethyl chitosan nanoparticle (CMCN) and poly (vinyl alcohol) (CMCN/PVA) onto the surface of modified bacterial cellulose sulfate (BCS) membranes via electrospinning as heparin-like substituent membrane. The similarity between heparin and heparin-like membranes (CPBS) is the presence of -SO_3,_ COO−, and -OH groups on the surface of the CPBS membrane. The CPBS membrane hydrophilicity was higher than the BC sulfate membrane, which could provide anticoagulant properties of the heparin-like membrane.

### 2.2. Drug Delivery Systems

Drug delivery systems are defined as a device or a formulation that is able to deliver an active substance to a target tissue to increase the efficiency of the active substance [98]. These DDS-based membranes have the ability to increase pharmacological activity, thereby reducing the side effects, increasing the solubility of the active substance, protecting the active substance from biodegradation and gradually releasing the active substance [99]. Additionally, to improve the drug release efficiency, different DDS sensitive to both external (magnetic, photothermal and electrical responsive) and internal stimuli (temperature, pH and redox responsive) have been developed. The first attempt to develop DDs was reported in 1950 in agriculture when they tried to develop systems for the controlled release of pesticides from a polymer matrix [100]. After that, this concept of the controlled release of the active substance was borrowed in the biomedical field [101]. In the past years, many biodegradable and bioabsorbable polymers were studied for the production of DDS. The release mechanism from a polymeric matrix is represented through drug diffusion, dissolution, and degradation of the carrier matrix [102]. Moreover, the usage of DDS leads to a decrease in the need for frequent administration of the active substance, leading to improved quality of life. The polymeric membranes used in DDS application require good stability during administration, biocompatibility, biodegradability and absorbability [103,104,105]. Depending on the size of the pores, the membranes can be classified into membranes with micropores (less than 2 nm), membranes with mesopores (between 2 and 50 nm) and membranes with macropores (more than 50 nm) [106]. Apart from the pores’ size, the pores’ shape is very important, so the pores of the membranes can have different shapes, such as cylindrical, conical or irregular. These morphological characteristics are very important when it comes to the development of DDS.

To obtain DDS-based membranes, several techniques are used such as phase separation technique, interfacial polymerization, stretching, ion-track etching, lithography and electrospinning [107,108]. In the ion-track etching method, a membrane can be obtained by irradiating a film with heavy ions forming ion tracks and leading to pore formation [108,109]. The films used for irradiation have been made of polymers, such as polycarbonate (PC), polyethylene terephthalate (PET), polyimide (PI), polypropylene (PP), polyethylene naphthalate (PEN) and also biodegradable polymers, such as polylactic acid (PLA), which are used as a polymer matrix to create track-etched membranes [110,111,112,113,114,115]. Ivanova et al. [115] reported the preparation of a biodegradable membrane based on PLA via the ion-track etching method. The pore size of the obtained biodegradable membrane was reported between 0.6 to 1.5 μm with a shape close to cylindrical. Further, it was observed that an increase in the etching time over 20 min shows a decrease in the roughness of both membrane sides. Lithography is another method for obtaining membranes. Through lithography, an ordered array of nano/micro pores on the surface can be obtained [108]. An example of the use of lithography in the developing of DDS is related by Patil et al. [116], who reported the well-defined pore formation on polyimide (PI), to be the support membrane for grafting poly(acrylic acid) (PAAc) hydrogel via free radical polymerization applying the same excimer laser. This type of DDS is pH-sensitive so at different pH levels (pH 7 and pH 3), it changes its permeability.

**Figure 3:**
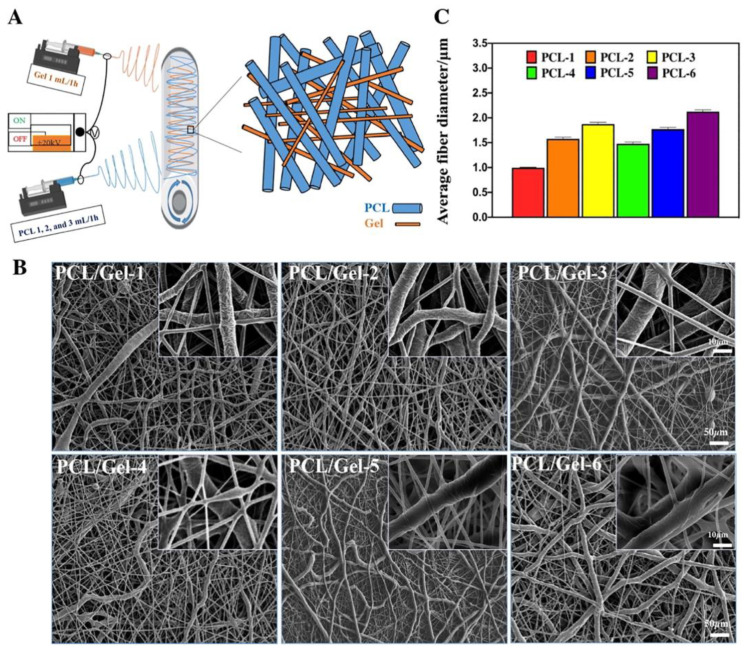
(**A**) Schematic illustration of the PCL/Gel membrane prepared by co-electrospinning technique; (**B**) SEM images of different PCL/Gel co-electrospinning membranes at various magnifications; and (**C**) the statistics of fiber size in different co-electrospinning membranes [123].

The electrospinning method is widely used in obtaining membranes with various applications in the medical field, such as in delivery systems and in tissue engineering, using the application of a high electric field to generate nanofibers from a charged polymer solution or melt [117,118]. It has been observed that if the electrospinning parameters are varied, such as polymer, solvent, polymer solution, processing parameters, and ambient conditions, different morphologies can be obtained in order to improve the mechanical strength and drug loading/releasing performance of membranes [119,120]. To obtain fibers and later membranes by electrospinning, both synthetic polymers can be used, such as polystyrene (PS), poly (vinylchloride) (PVC), and PLA, as well as natural polymers, such as silk fibroin, fibrinogens, dextran, chitin, chitosan, alginate, collagen, and gelatin [120]. Ren et al. [121] reported an electrospun membrane for controlled drug delivery for the acceleration of diabetic wound healing based on PLA and dimethyloxalylglycine (DMOG)-loaded mesoporous silica nanoparticles (DS). All the membranes showed well-organized topological structures, with ellipsoidal-shaped nanopores, which were arranged with the major axis along the fiber direction. Co-electrospinning method, also known as coaxial electrospinning, is a method derived from electrospinning, the difference being that in the case of co-electrospinning, two needles are used instead of one needle, which makes it possible to obtain a core-shell nanofiber [122]. Al-Badani et al. [123] reported a possible polycaprolactone/gelatin membrane as a tunable drug delivery system for bone tissue regeneration via co-electrospinning (Figure 3). It was observed that by adjusting the poly(ε-caprolactone) (PCL) fibers, the degradation of gelatin and the release profile of hydrophilic drugs/proteins could be effectively controlled.

The phase separation technique is one of the simplest methods for obtaining membranes. Phase separation can occur when the thermodynamic quality of a polymer solution usually decreases when the temperature is decreased, and by immersion precipitation [124]. Zeinali et al. [125] reported Poly(butylene succinate) (PBS) membrane via thermally-induced phase separation (TIPS) for Curcumin (CUR) and piperine (PIP) release. Additionally, it was reported that the variation of solvent system, thermal gradient and cooling direction changed the pores’ architecture, which could affect the release profile. The interfacial polymerization method is used for membrane formation, which is one of the most important processes in commercial fabrication [126]. In this method, the polymer solution and the solvent are poured onto a flat surface to form a film. After that, the controlled process of exchanging solvent for a nonsolvent takes place, leading to a membrane formation [127]. Ding et al. [128] described an antibacterial and anti-inflammatory membrane based on porphyrin-covalent organic frameworks (COF) with encapsulated ibuprofen (IBU) via an in situ interfacial polymerization and impregnation approach. The reported membrane displayed high O_2_ generation and controllable ibuprofen release at body temperature for wound healing. 

Generally, the surface modification of membranes is carried out for better biocompatibility and biodegradability [108]. The biocompatibility of the membrane is represented by it is represented by the response to the interaction with biological fluids, without triggering any kind of immune response or infection. The most used techniques for membrane surface modification are blending [129], grafting [130] and plasma initiation [131]. The blending surface modification method is one of the most convenient methods due to reduced cost. Liang et al. [132] proposed the improvement of the blending strategy for membrane modification via surface segregation using surface-tailored amphiphilic nanoparticles. After the functionalization, the membranes achieved a notably increased blending efficiency, resulting in a dramatically enhanced surface hydrophilicity. Parveen et al. [133] reported chitosan/PEG blended PLGA nanoparticles for cancer drug delivery. The functionalization with PLGA decreases the aggregation due to the repulsion of serum proteins, leading to very good stability for days and the lowest percentage of uptake by macrophages. Surface modification of polymeric membranes via grafting has become a very popular method in recent years for preparing a “tailored” membrane surface with desired functions [134]. For example, through this method of functionalization, membranes sensitive to pH variation can be obtained only by grafting different functional groups or such polymers onto the surface of the membrane [135]. Surface modification by grafting can be achieved through several techniques, such as grafting through light [136,137], grating via thermal treatment [138,139], grafting polymerization through plasma irradiation [140,141], atom transfer radical polymerization (ATRP) surface initiated method [75,142,143,144], reversible addition fragmentation chain transfer (RAFT) polymerization [145,146,147] and redox reactions grafting [148]. 

In the modification of the membrane surface with plasma treatment, plasma is a confined ionized gas obtained from the dissociation of gas-forming plasma after applying an electric field in the reactor [149]. The plasma effect on the membrane occurs only on the surface, not affecting the properties of the bulk [150]. The plasma treatment can introduce chemically reactive functional groups onto polymer substrates to increase biodegradability or biocompatibility [151]. For example, an O_2_ plasma is able to generate ─OH radicals on the membrane surface, leading to an influence on the hydrophilicity of the membrane surface [152]. 

Further, the hydrophobic or hydrophilic character of the membrane can have a say in increasing biocompatibility, so hydrophilic membranes are preferred for biomedical applications as they improve the flux through the membrane and fouling properties [131]. The degree of hydrophilicity or wettability of the membrane is one of the most important parameters to control cell behavior through protein adsorption [153]. However, the hydrophilic/hydrophobic character of the active substance is a criterion to be taken into account, so that the functionalization of the membrane surface can improve the interaction between the membrane and the active substance. Hardin et al. [154] shared the functionalization of polyacrylic acid membrane for potential drug delivery application via condensation reaction with N-(3-dimethylaminopropyl)-N-ethylcarbodiimide hydrochloride as our coupling reagent. It was shown that the choice of the hydrophobic group has an effect on the polymer solubilization properties. Sagitha et al. [155] reported the functionalization of the polyurethane membrane with β-Cyclodextrin, leading to increased hydrophilicity and thermal stability. The addition of β-Cyclodextrin provides a higher vapor transmission rate, hemocompatibility and cell viability for functionalized membrane. Moreover, the obtained β-Cyclodextrin- polyurethane membrane was functionalized with nanochitosan for better biocompatibility.

Stimuli-responsive membranes have attracted attention in recent years due to their extraordinary properties such as the ability to change their physicochemical properties in response to changes in their environment [156,157,158]. These stimuli-responsive membranes are able to respond to changes in pH, temperature, light and magnetic fields, being able to release various therapeutic agents in a targeted and gradual manner [157]. For example, pH-responsive membranes are obtained from polymers with ionizable acidic/basic residues which can be employed in drug delivery applications, and this is possible due to the variation of a normal pH in the body and also the variation in pH caused by different conditions, such as inflammation, infection and cancer [159,160]. Notably, pH-responsive membranes are able to target a specific area in the body and release their active agent with an increased therapeutic impact and reducing the side-effects [161]. These pH variations can result in the modification of crosslinking processes so that the protonation or deprotonation of acidic/basic groups can generate distinct interactions between a therapeutic agent and a material, resulting in the release of the active substance [159]. Additionally, a very prominent example of pH-responsive DDS membranes is based on the alginate-chitosan complex, due to advantageous properties such as biocompatibility, biodegradability, pH sensitivity, and mucoadhesive property [162]. Additionally, the protonation of the amino groups leads to the polymeric backbone of chitosan becoming charged, resulting in charge repulsion and swelling [163]. Further, due to their opposite charged polymeric backbones of chitosan and alginate, polyelectrolyte complexes are obtained [164]. Schoeller et al. [163] reported the deposition of chitosan and alginate layers forming polyelectrolyte complex through layer-by-layer assembly on Poly(lactic-co-glycolic acid ) (PLGA) nanofibers to introduce pH-sensitivity for the controlled release of ibuprofen. The results show the inhibition of ibuprofen release at an acidic pH due to the pH-sensitive membrane suggesting that the obtained membrane protects the active substance in the acidic environment of the stomach, leading to an improved release at higher pH values without an early release and reduction in drug concentrations.

Temperature-responsive membranes for DDS are obtained from temperature-sensitive polymers with the property of sol-gel transitions above a certain temperature [165]. The temperature-responsive polymeric membranes are able to change their phase below the lower critical solution temperature (LCST), leading to the hydrogen bonds between the polymer and water molecules, allowing the polymer to swell and release the active substance [166]. The magnetic-responsive membranes are usually filled with magnetic-active nanoparticles of metals, metal oxides, or ferromagnetic materials which under the action of electromagnetic radiation can release the active substance in a targeted manner and enhance drug accumulation at the sites [167,168,169]. Photo-responsive membranes are able to release drugs using light sources, such as ultraviolet (UV), visible, and near-infrared (NIR) light [34]. The permeability of the photo-responsive membrane is influenced by switching irradiation between ultraviolet light (UV) and green light (Vis) [170]. The photo-responsiveness property is ensured by the presence of different photo-reactive groups, such as azobenzene, triphenylmethane and spiropyran groups in the polymer matrices by entrapping, cross-linking, and introducing them as a side chain or part of the main chain of the polymeric matrix [171]. Other physical characteristics, besides stimuli-responsiveness, which are studied and discussed in the DDS field are particle size distribution and polydispersity index (PDI) and length of polymers [172,173]. The PDI is an important physical characteristic to be considered in DDS development due to its influence on stability, drug release profile and drug penetration [172,174]. The length of the polymer can influence the release of the active substance from the DDS-based membrane by increasing the release rate as the polymer length decreases [173]. 

Further, opsonization is an essential characteristic of biological barriers in order to control drug delivery so that the macrophage cells identify the presence of the membrane-based release system as a foreign body and remove them to protect the body [175,176]. A method to combat opsonization is represented by PEGylation to prevent phagocytosis [176]. PEGylation is used to increase the efficiency of drug and gene delivery and decrease the immunogenicity of the proteins without significantly affecting the activity [177]. The molecular weight (MW) of the PEG link can influence the internalization so that an increase in MW leads to the increased flexibility of the PEG chain, leading to the entanglement of PEG chains and reducing the binding of the ligand to the receiver [178].

### 2.3. Membrane Oxygenator

**Figure 4:**
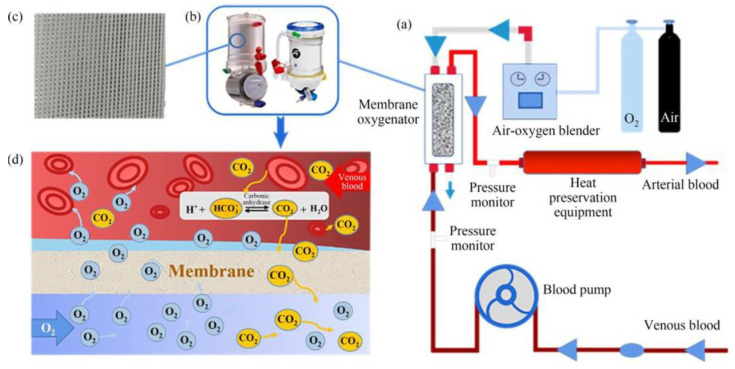
(**a**) ECMO system composition diagram; (**b**) Schematic diagram of common membrane oxygenators; (**c**) hollow fiber membrane filament for gas and blood exchange; (**d**) Schematic diagram of blood oxygen exchange principle. (reproduced with permission after [26]).

Recently, due to the COVID-19 pandemic, the need to develop artificial oxygenators is growing. As the name implies, oxygenators are defined as medical devices for respiratory support. An example of an oxygenator is represented by extracorporeal membrane oxygenation (ECMO), which is used in the treatment of severely ill patients with cardiopulmonary impairment caused by infectious diseases [179]. The general principle of the operation of oxygenators is that when the blood passes through the oxygenator, the oxygen level increases, and the CO_2_ level decreases so that the non-oxygenated blood is oxygenated (Figure 4) [26,180,181]. The extracorporeal membrane oxygenators are they are made up of a pump that has the role of pumping the blood and the oxygenating membrane that has the role of oxygenating the blood [179,182,183]. The most common causes of complications in patients using an oxygenator are clotting and bleeding [184]. The material from which the oxygenating membrane is obtained must have high permeability, high mechanical strength, free defects, high biocompatibility and hemocompatibility [179,185]. For example, in the case of hemodialysis, membranes are completely sterile and the whole tubes used for extracorporeal circulation of blood are also sterile in order to prevent sepsis. Normally, in order to avoid the formation of clots in the oxygenation installation, they administer anticoagulant drugs, such as heparin. Unfortunately, this method has the disadvantage of the possibility of severe bleeding [186,187,188,189]. Another limitation of the ECMO system is the adherence of various biomolecules on the surface, which can be overcome by incorporating small molecules, such as heparin, in the coating of the ECMO system surface [190]. The anaphylatoxins are attached to the biomaterial surface and have numerous implications in hemostasis. The polymers under the ECMO system have an effect on the CR3 granulocyte receptor, resulting in the adhesion of principal neutrophils and monocytes [190].

Hollow fiber membranes have been used as an oxygenator and are usually obtained via a phase inversion process [191,192]. The commonly used polymers for hollow fiber membranes are hydrophobic polymers, such as polymethylpentene (PMP), polypropylene (PP), PDMS, polysulfone (PSf), polyethersulfone (PES), polyethylene (PE) and polyvinylidene fluoride (PVDF) [26,192,193,194,195,196,197,198]. Wang et al. [194] reported the production of poly (4-methyl-1-pentene)/polypropylene (PMP/PP) thin film composite (TFC) with a PVA/PSS coating was anchored on the membrane surface via crosslinking and PDA binding for membrane oxygenator application. PMP is used in oxygenator membrane production due to increased gas exchange properties, low diffusion resistance and absent plasma leakages. After surface modification of membranes showed excellent hydrophilicity and coagulant factors, adsorption capability, which significantly inhibits the activation of platelets and the adhesion of proteins, thus blocking the blood coagulation due to increased sulfonate groups. Park et al. [199] proposed the fabrication of functionalized poly(vinylidene-co-hexafluoropropylene) or PVDF-co-HFP coated, using Hyflon AD60X to minimize pore wetting and interactions with blood. These functionalized membranes have very low protein adsorption and a high contact angle for both water and blood, leading to improved hemocompatibility. 

### 2.4. Artificial Liver

**Figure 5:**
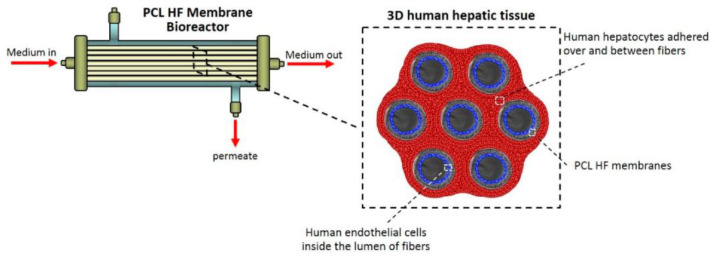
Poly(ε-caprolactone) (PCL) hollow fiber (HF) membrane bioreactor and scheme of the 3D human hepatic tissue realized by culturing human hepatocytes over and between PCL HF membranes parallel assembled at a distance of 250 µm, and endothelial cells compartmentalized in the lumen of the fibers. The cells were in communication through the porous wall of the membranes (reproduced with permission after [204]).

Liver disease is a worldwide major public health problem, being one of the most life-threatening diseases caused by obesity, non-alcoholic fatty liver disease, high alcohol consumption, hepatitis B or C infection, autoimmune diseases, cholestatic diseases, and iron or copper overload [200]. Chronic liver diseases (CLDs) are more liver-related diseases characterized by a decreased hepatic function as a result of chronic inflammation of the liver, leading to the development of cirrhosis [201]. The only established successful treatment for end-stage liver disease is liver transplantation [202]. The drawback of liver transplantation is organ shortage [203]. As a solution to the transplant limitation, researchers have tried to develop an artificial liver able to provide detoxification, synthesis, biotransformation and excretion functionality as performed by the liver [202]. Various polymeric membranes have been reported in order to develop an artificial liver [25,204,205]. The developed membranes are able to mimic the physiological environment. Further, in the last years, the development of membrane bioreactors was reported, which are able to potentially optimize the treatment during reversible acute liver disease or while waiting for a liver transplant [206]. The hollow fiber membrane bioreactors (HFMBRs) are used to develop liver tissue constructs for bioartificial liver (BAL) or as an in vitro drug discovery and testing platform [207]. Salerno et al. [204] developed biodegradable hollow fiber (HF) membranes of poly(ε-caprolactone) (PCL) with permeability, structural and mechanical properties that supported the cell adhesion and functionality (Figure 5). The endothelial cells were cultured in the lumen of the fibers, and hepatocytes in the shells of the fibers, communicating through their secreted molecules that permeate into the microporous structure of the HFs membrane wall. The sustaining glucose consumption, albumin synthesis, urea production and drug biotransformation function were sustained for 18 days by the hepatic tissue. The biocompatibility of PCL was studied by Slivac et al. [208] where PCL was used as a matrix for electrospun PCL Mats for tissue scaffolds for hepatic cell application. The hepatic cells are grown and attached on obtained scaffolds, yet the PCL scaffolds are able to mimic the bioactivity found in the original tissue matrix. Teotia et al. [25] presented a hollow fiber membrane based on asymmetric porous polysulfones and polysulfone-Tocopheryl polyethylene glycol succinate (PsfTPGS) composite via phase inversion procedure and subsequently surface modified with chitosan using sulfonation with concentrated sulfuric acid. The addition of chitosan confers biocompatibility to the obtained composite membranes due to structural similarity to glycosaminoglycans, a native liver Extracellular matrix (ECM) component leading to supporting cell growth, proliferation and the expression of the liver-specific function. 

### 2.5. Artificial Pancreas

The pancreas is an internal organ located in the abdominal cavity just behind the stomach with a role in the regulation of body metabolism [209]. The function of the pancreas is to control glucose homeostasis through the secretion of endocrine hormones, such as insulin, and to produce exocrine enzymes required for digestion [210]. In type 1 diabetes, patients are unable to produce insulin due to an autoimmune response to the body’s insulin-producing beta cells. As a result, they need insulin treatment [211]. In the case of type 2 diabetes, the organism develops insulin resistance, and the glucose level remains high. The purpose of the artificial pancreas is to protect the pancreatic islets from the immune system’s response, allowing the transfer of insulin, oxygen and other nutrients [212]. The encapsulation of islets could be intravascular or extravascular, depending on the position of the implant. Over the last 40 years, were studied different bio-artificial pancreas (BAP) devices [213]. The semipermeable membranes for intravascular encapsulation are obtained from synthetic polymers, such as polyacrylonitrile-polyvinylchloride (PAN-PVC) copolymer, polycarbonate, ethylene vinyl alcohol copolymer (EVAL) fibers, poly-amino-urethane, nonwoven polytetrafluoroethylene (PTFE) fabric and nylon [214]. These membranes must fulfill several characteristics, so that they can be used to protect and segregate the islet cell from the immune response, allowing the oxygen, glucose, and other nutrients to permeate so that the level of glucose in the blood could be controlled. Further, these membranes must present a high hemocompatibility and biocompatibility, and they must be easy to implant so that, in case of failure, can be replaced very easily [214]. 

### 2.6. Osteosynthesis Membrane

Another application of membranes in the medical field is represented by tissue engineering, or more precisely, osteosynthesis and bone regeneration. The research of materials with applicability in bone regeneration started from the need to replace the materials from which metal implants were obtained in order to fix fractures. The major disadvantage of these materials was that after implantation, followed by fracture healing; another surgery was performed to remove the metal implants. Following this implant extraction operation, there is a slight possibility of the apparition of infection, removal problems of jammed implants, implant migration and associated extra health care costs. The solution was to develop new polymeric materials capable of fixing bone defects, as did metal implants, but with the ability to biodegrade at the same time as the actual healing and to facilitate bone healing [215]. Biodegradable materials are defined as materials that, after a period following implantation in the body, are disintegrating [216]. An example of biodegradable materials can be constituted some polymer materials which may disintegrate *in vivo* via the effect of the biological environment on the integrity of the material, leading to surface erosion or bulk erosion [215]. An osteoconductive material is a material able to serve as a scaffold onto which bone cells, such as osteoblasts and osteoclasts, can attach, migrate, grow and/or divide [217]. The main properties of biodegradable materials for osteosynthesis are excellent mechanical properties, control over degradation time, and biocompatibility [218]. Generally, membranes for osteosynthesis are made of biopolymers from natural sources, such as collagen, chitosan and cellulose, and synthetic sources, such as expanded polytetrafluoroethylene (e-PTFE), poly lactic acid (PLA), and polycaprolactone (PCL) [219,220,221]. Synthetic polymers are more susceptible to an inflammatory response in comparison with natural polymers. Still, an advantage worth mentioning is the capability to control biodegradability, processability, and drug-encapsulating ability [219]. Some of the most used biopolymers in osteosynthesis membranes are chitosan, collagen and cellulose due to increased biocompatibility through a lower immune response and osteoblastic adhesion [222,223,224]. The limitation of collagen is that if collagen is of animal origin, it could transmit disease from animal to human and also, the mechanical properties are lower [225]. In order to increase the mechanical properties, it was reported that collagen crosslinked with glutaraldehyde [226]. Unfortunately, glutaraldehyde shows cytotoxicity due to by-product degradation in later metabolic pathways [227]. As a result, cross-linking materials have been studied, such as EDC(1-ethyl-3-(3-dimethylaminopropyl) carbodiimide)-NHS(N-hydroxysuccinimide), genipin and oligomeric proanthocyanidins (OPCs) [221,226,228]. Liu et al. [229] used genipin, which is a natural cross-linker for collagen type 1 immobilization on Ti surfaces for improvement of the ensuing biological responses. 

**Figure 6:**
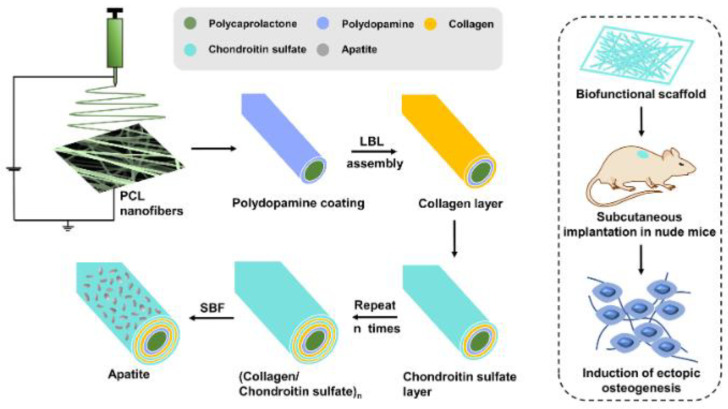
Schematic of the biofunctional bone scaffold fabrication with LBL self-assembly on electrospun fiber membranes followed by apatite deposition (reproduced with permission after [230]).

Further, many researchers are adding hydroxy apatite for an additional increase in osteosynthesis. Zheng et al. [230] presented a multilayer membrane based on collagen/chondroitin sulfate (COL/CS) which was assembled onto an electrospun PCL membrane followed by apatite mineralization (Figure 6). It was shown that the multilayer membrane has favorable mechanical properties, hydrophilicity, biodegradability, and outstanding biocompatibility. It also showed that the multilayer membrane promotes proliferation and osteogenic differentiation due to the addition of the apatite, which is able to promote bone-implant bonding and osteoinduction. The osteoconductivity of the membrane could be used as adjuvant films for osteointegration of a metallic implant, leading to better compatibility of the implant, as the membrane is placed between the implant and the bone. Pandele et al. [20] proposed polylactic acid and micro-structured hydroxyapatite particles in order to obtain composite films. The addition of hydroxyapatite doesn’t affect the degradation temperature, but a decrease in the crystallinity of the composite films was observed.

**Figure 7:**
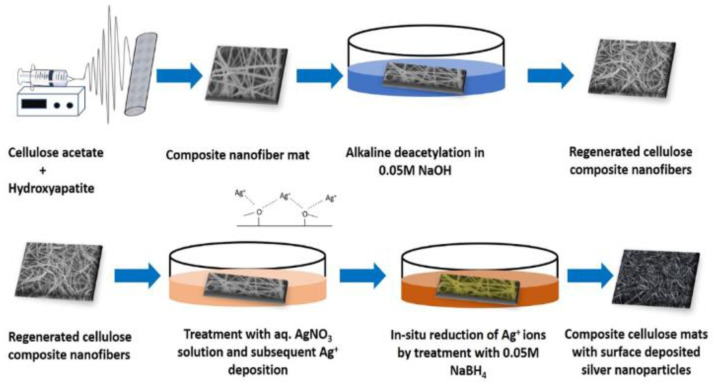
Schematic representation of the fabrication of regenerated cellulose nanofiber mats containing HAp and Ag NPs. Sequential steps are shown to describe the various process involved in the fabrication process (reproduced with permission after [235]).

Cellulose acetate is a cellulose-derivate biopolymer extremely used in membrane development for osteosynthesis membranes due to its biocompatibility, biodegradability and hydrophilicity properties [231,232,233]. Sofi et al. [234] described the development of a scaffold for tissue engineering applications based on cellulose acetate functionalized with hydroxyapatite and silver nanoparticles (Figure 7). The mineralization with hydroxyapatite gives an environment for adhesion, growth, and proliferation of chicken embryo fibroblasts, thus with more hydroxyapatite content which leads to better cell proliferation. The addition of silver nanoparticles confers antimicrobial properties to the composite’s membrane. 

**Figure 8:**
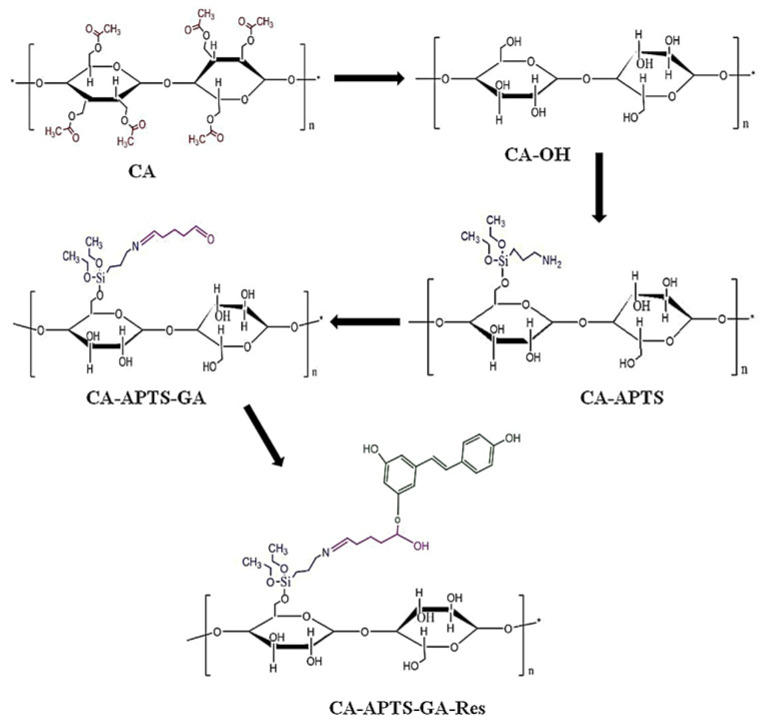
Schematic representation of the reaction sequence for the derivatization of cellulose acetate membranes with resveratrol (reproduced with permission after [238]).

Besides functionalization with hydroxyapatite for better osteosynthesis of the membranes, resveratrol is also utilized for a stimulatory effect on bone formation [234,236]. Resveratrol is a polyphenol compound present in fruits and vegetables with great antioxidant properties, antitumoral and antibacterial properties, and also it was reported that it might control cell proliferation [237]. Pandele et al. [238] described the functionalization of cellulose acetate membrane with resveratrol using aminopropyl triethoxysilane (APTS) and glutaraldehyde as linker molecules in order to improve osteointegration (Figure 8). The results showed that the resveratrol functionalization confers osteoblasts viability and differentiation potential in terms of alkaline phosphatase (ALP) activity, which is present in a large amount in the cells of mineralized tissue and bone mineralization and is essential to the formation of hard tissue [239]. 

A limitation in osteosynthesis application is represented by poor bonding strength between bioceramics and biopolymers due to their dissimilarity in physical and chemical properties, leading to poor bonding strength between the two phases [240]. As a solution to this limitation, the use of coupling agents has been reported with two-parent groups (hydrophilic and hydrophobic) can improve the interface between the polymer and ceramic material, thus improving the properties [241,242,243]. As mentioned earlier, these coupling agents are able to help the interaction between the polymer matrix and the ceramic matrix through two different functional groups, one of which can react with organic molecules, and the other can absorb inorganic surfaces in order to obtain a firm bond [240]. An example of a coupling agent often used is represented by Amino-propyl-triethoxy-silane (APTES) used as a surface modifier for glass and ceramic in order to increase the strength properties of biodegradable composites, as well as to improve the adhesion of the material to tissues [35,244,245]. Biernat et al. [245] reported the functionalization of porous calcium phosphate ceramics (Ca-P)/ poly(L-lactide) (PLA) composites with APTES and alendronate in order to improve the mechanical properties and cytocompatibility. After the functionalization with APTES, an increase in mechanical properties of the composites and an improvement in biocompatibility were observed through Sodium alendronate binds to hydroxyapatites in the bone.

### 2.7. Membranes for Sensors

Sensors and biosensors based on polymeric membranes used in biomedical applications mainly use three types of membranes – functionalized, molecularly imprinted and composite. The functionalized membranes offer the possibility of immobilization on the surface of the various species that interact with the analyte of interest, the most important being the enzymes for the detection of various organic species [246] or molecules with complexing capacity for the detection of ions (such as crown ethers) [247]. The detection system involves both the use of electrodes and complex structures such as Surface Acoustic Wave (SAW) platforms [248]. Urease immobilized onto poly(vinyl alcohol) was used as an indicator electrode for the urea sensor with a detection limit in the range of 1 × 10^−5^–5 × 10^−4^ M and a sensitivity of 19,069 mV/decade [249]. A sensor for triglyceride detection was developed by immobilization of lipase on a polyethyleneimine film (using glutaraldehyde as a linker molecule) deposited on a glassy carbon electrode. The sensor showed a detection limit in the range of 100–500 mg/dL with average recovery values from 95.47 % to 101.05 % [250]. Introduced by Wulff in 1972 [251], molecularly imprinted polymers found their applicability in a wide range of biomedical applications, such as the controlled release of drugs or even sensors and biosensors [252] due to the remarkable properties of molecular or even species with larger dimensions recognition. Thus, molecularly imprinted membranes were used for the detection of cells [253], DNA fragments [252], various saccharides present in the urine [254], proteins [255] or genes [256]. The detection systems used are based either on Quartz Crystal Microbalance (QCM) [257] or the volumetric principle detection [258]. The simultaneous determination of cholesterol and cholestanol was performed with a sensor based on molecularly imprinted polymer membranes obtained from methacrylic acid, ethylene glycolmethacrylate, 2,2-dimetthoxy-2-phenyliacetonephenon. To ensure the electronic conductivity of the entire sensitive structure, the membranes incorporated carbon nanotubes (MWCNTs) and were deposited on the screen-printed carbon electrode with gold nanoparticles [259]. The sensor has been tested both in terms of signal accuracy from simulated solutions and complex matrices, as well as from the point of view of stability in time, at repeated measurements of 8 times a day, maintaining its stability and accuracy for 45 days. Qualitative and quantitative detection of lysozyme was successfully performed using a biosensor from molecularly imprinted membranes embedded with l-cysteine-capped Mn^2+^-doped ZnS quantum dots [260]. The novelty of the method lies in the possibility of using the optical signal (much more precise than the electronic one), due to the fluorescence effect of quantum dots. The sensor presented linear detection ranged from 1.0 × 10^−7^ to 1.0 × 10^−6^ mol L^−1^ with the detection limit of 10.2 nM. The simultaneous detection of three chemical species - insulin, proinsulin and C-peptides, was achieved by mixing those species with N-methacryloyl-(L) 3-histidine methyl ester, 2-hydroxyethyl methacrylate and ethylene glycol dimethacrylate, followed by the polymerization of the precursors under UV at the surface of the electrode (thick-film boron doped diamond electrode) [261]. The detection limit was in the range of 1–16 pM for insulin, 4–25 pM for proinsulin and 8–88 pM for C-peptide, respectively, in both artificial and real human serum samples. The composite membranes used in the field of biosensors are mainly based on the use as a filler of carbon nanotubes or graphene functionalized with enzyme specific for a certain biological species, the polymer representing usually the matrix that protects the sensitive part and ensures the deposition on different electrodes [262].

## 3. Conclusions and Future Perspectives

The field of polymer membranes for biomedical applications is one in continuous development, and the challenges that must be solved contribute to the dynamics of this field. The future perspectives are equally addressed to all the applications that membrane materials have in relation to this field. Some of them will be presented below. In the field of hemodialysis, it is unlikely that polysulfone will be replaced too soon. Instead, the process will be able to be combined, in particular with the controlled release of drugs in order to make especially efficient the treatment of diseases derived from chronic renal dysfunction, such as liver cancer, for example. The synthesis of supramolecular architectures that can download the drug under the action of the tumor marker - alpha-fetoprotein. Additionally, in the field of membranes that combine two therapeutic procedures, the development of membranes that treat diabetes, either by releasing insulin or by releasing other active substances used in the treatment of this medical condition, would be equally useful. Another challenge in the field of hemodialysis is the easy development of membranes, especially custom-made for ‘one-day’ hemodialysis processes to remove compounds that have reached the body as a result of various intoxications. The retention of heavy metals [263,264] from the blood or various organic compounds (pesticides, overdoses of medicines or drugs) could be achieved by filtering the blood with the help of the one-day hemodialysis procedure. The future of hemodialysis depends on a combination of the current membranes, as we know them at the moment, and microfluidics. Devices are to be worn permanently by the patient and to filter the blood with membranes that can be changed every two to three days. In the field of artificial liver and artificial pancreas applications, cell viability still presents a problem. The use of hepatocytes of porcine origin or Langerhans cells is limited by the extremely short time in which they maintain their viability inside the membranes used. Finding solutions to condition the membranes or to ‘freeze’ them immediately after obtaining them would make possible the sustainable use of these therapeutic solutions. Related to polymers used for these applications, most probably due to economic and environmental reasons, we will assist in a transition from synthetic polymers to natural ones. Cellulose and its derivatives are the most promising candidates, especially for osseointegration, but also for hemodialysis or drug delivery. The main advantage is given not only by the fact that it is a natural source polymer and presents high biocompatibility but also by the fact that is bioresorbable due to its chemical structure. The only residue resulting from decomposition is glucose.

## Data Availability

Not applicable.
